# Moving Along and Beyond the Spectrum: Creative Group Therapy for Children With Autism

**DOI:** 10.3389/fpsyg.2019.00417

**Published:** 2019-03-12

**Authors:** Sharon Vaisvaser

**Affiliations:** ^1^ The Academic College of Society and the Arts (ASA), Netanya, Israel; ^2^ The Autism Research and Treatment Center, The Association for Children at Risk, Giv’at Shmuel, Israel

**Keywords:** autism spectrum disorder, group therapy, play, relational movement, sensory motor experiences, spontaneous communication

## Abstract

Group therapy for autism confronts the core of the syndrome. Non-directed dynamic approaches, in which moment-to-moment spontaneous expressions drive the content of group sessions, are even more intricate. The implementation of nonverbal creative techniques holds the key to self-expression and self-other exploration, promoting communication and play. This manuscript offers an integrative conceptual model and a case report regarding such mind–body therapeutic perspective. The creative arts intervention is presented *via* a small group of young minimally verbal children with autism, deprived of communicative language, offering an interdisciplinary perspective to delineate group challenges and rationale, process, and outcomes. Vignettes are provided to illustrate the group development. A thorough discussion follows, addressing three intertwining axes: firstly, the implications of nonverbal creative means are considered; secondly, the psychophysiological processes set in motion through sensory-motor experiences are deliberated; and thirdly, the emergence of “moments of meeting” and spontaneously generated playful group activities are enlightened.

## Introduction

Therapeutic creative group work for autism spectrum disorder (ASD), a diverse and complex neurodevelopmental condition, confronts the two broad categories of symptoms, as mentioned in the DSM-5 (American Psychiatric Association, 2013). These include deficits in social–emotional reciprocity, impairments in both verbal and nonverbal communicative exchange, and modulation of behaviors according to social context, with poor ability to develop, preserve, and understand relationships (Criterion A). Limited spontaneous initiation of social interactions relates to the severity of symptoms. The second cluster of symptoms includes narrow and fixed fields of interest, rituality, and rigid repetitive patterns of behavior, including sensory hyper- and hypo-sensitivities (Criterion B). These symptoms appear in early development (Criterion C) and cause significant impairment in daily function (Criterion D).

Characterized by heterogeneous manifestations, the high variability in expression and severity of symptoms among individuals with ASD, with diversity at the behavioral, neurocognitive, and genetic levels (Happé et al., 2006; Lenroot and Yeung, 2013; Just et al., 2014), deepens the challenge of conducting group therapy. Diverse abnormalities were also shown with regard to sensory and motor aspects (Haswell et al., 2009; Fournier et al., 2010; Shetreat-Klein et al., 2014; Hannant et al., 2016), suggested to have crucial impact on social function (Donnellan et al., 2013).

From a psychodynamic perspective, the autistic phenomena is attributed to impaired psychophysical infrastructure, rooted in an excessive need for symbiotic merging and the mental registration of separation as trauma (Tustin, 1992). Thus, social contexts trigger anxiety and an atypical sensory auto-stimulation develops in order to deviate away from human contact. The motivation for connection is contradictory to the autistic state, characterized by a need for sameness and an attack on the natural tendency for integration between the functions of movement-sensation-affect-cognition, along with connection and separation (Pollak, 2017). The ritualistic autistic actions aggravate deficits in orienting to novelty rather than preservation and are therefore opposite to play, which is based on openness and curiosity. Therapeutic constructs employing fantasy are not easily applicable, play is frequently empty of excitement, and narrative development tends to be repetitive and without psychic salience (Shapiro, 2009).

Well-structured group interventions for ASD are common, promoting acquisition of predefined developmental and social skills, adaptive behaviors, and linguistic abilities. Due to the need for predictability, difficulties of individuals with ASD are more pronounced in unstructured dynamic settings (Volkmar et al., 2004). Poor spontaneity was suggested as a major contributor to the impoverished social behavior, as they do not readily engage in instinctive affect exchanges with others (Trevarthen and Aitken, 2001). They lack spontaneous attention and self-induced exploration of social encounters, and have impaired capacity to efficiently enact desired intentions through actions of the body (Chiang and Carter, 2008; Chevallier et al., 2012; Kohls et al., 2012; Trevarthen and Delafield-Butt, 2013). These point to the importance of the non-directed social environments that prompt spontaneous interpersonal action.

Alvarez (2004), in line with Hobson’s notion of the “spared function” in autism and Bion’s notion of the “non psychotic” part of the personality, highlights the importance of making contact with “non autistic” parts through a relational and developmental approach. This is strengthened by the novel concept of “neurodiversity” (Baron-Cohen, 2017). The emphasis on the bodily “lived experience,” promoting intra- and interpersonal development through engagement in interactive perceptuo-motor experiences, was suggested to correspond with and support ASD broad atypicalities (Donnellan et al., 2013). Furthermore, therapeutic focus on relational movement coincides with the well-recognized role of the mirror neuron systems (MNSs) (Gallese et al., 2009; Rizzolatti and Sinigaglia, 2016) and its abnormalities implicated in autistic social deficits (Enticott et al., 2012; Gallese et al., 2013; Casartelli et al., 2016).

Indeed, it is the nonverbal aspects of social interaction and embodied relational experiences that lay the foundation for the development of brain function (Hari et al., 2015). This concurs with the neuroscientific framework of an “enactive mind” that views cognition as bodily experiences upon encountering salient aspects of the environment, an approach discussed both in general (Goldman and de Vignemont, 2009) and specifically in relation to ASD (Klin et al., 2003; De Jaegher, 2013). Motor movement was recognized as a means for information gathering, communication, and construction of cognitive schemes of the self in relation to others (Shahar-Levy, 2009; Koziol et al., 2012). Joint action, bodily coordination, and mutual imitation have been emphasized as a body-based way of instantiating a socioemotional connection with another (Marsh et al., 2009). Creative and imitative activities, which employ nonverbal forms of expressive movement, may prompt development of intentional and rhythmic emotional expressions, as fundamental components of shared narrative sequences and social understanding that appear to be disrupted in autism (Trevarthen and Delafield-Butt, 2013; Delafield-Butt and Trevarthen, 2015).

Dance/movement therapy, defined as “the psychotherapeutic use of movement to further the emotional, cognitive, physical and social integration of the individual” (American Dance Therapy Association, 2009), was shown to support interpersonal relating and attentive behavior of young children with autism (Hartshorn et al., 2001; Tortora, 2005; Devereaux, 2012; Samaritter, 2017). This animated active form of psychotherapy may contribute to increased awareness of self and others and improved body image, through exploration and process-oriented learning, further enabling children with ASD to expand their movement repertoire (Scharoun et al., 2014). The inclusion of visual art encompasses work on a sensory level that can help children who are overwhelmed by typical sensory input (Dougherty et al., 2016). Furthermore, compatible use of art materials may support creativity and imagination and provide a product to focus on beyond the process (Martin, 2009).

Such conceptions drove the rationale for the presented group model, based on dynamic socioemotional processes brought into action in the interactional here-and-now. The current group was conducted in an ASD kindergarten, co-led by a dance\movement therapist (the author) and an occupational therapist and included three minimally verbal autistic children, diagnosed as low-functioning. We were motivated to support primary creativity in these nonspeaking children, in a safe and open atmosphere, increasing tolerance for uncertainty vital for the development of play (Winnicott, 1971; De Astis, 1997). The core of the meetings was non-structured, with an anchor of structured opening and closing songs and the use of particular creative props. The body of the group included a phase of non-directed drawing with oil pastels on paper and a phase of free movement with stretch bands, an elastic cloth large enough to accommodate the group. Sessions were accompanied by consistent instrumental music with various tempos. Props were used as a means of expression and a bridge for connection, enabling the children to tolerate interactions that might be too threatening if carried out more directly (Erfer, 1995).

The group goal was to establish a social environment that shapes the group in an ongoing manner, thus allowing the development of autonomous creative forces, enabling softening of the protective autistic shell. We aimed to enhance perceptual awareness of self and others’ intentions, conveyed through creative modalities involving sensorimotor experiences. We further intended to promote the choice of engagement in communicative actions, enhancing mimicking, spontaneity, and emergence of social reciprocity.

## Background—Description of the Group

### Group Participants

The presented group consisted of three boys (ages 4.5–6, referred to as “Child A/B/C”). Ethical approval was not required for this study as per institutional and national guidelines as the group reflected usual clinical practice within the service. Written informed consent was obtained from the parents of all participants. The clinical descriptions of the group and participants have been anonymized so that neither the location nor the individual participants could be identified. Each child had unique physical, developmental, and behavioral manifestations. Despite heterogeneity among group participants, unifying factors were the impaired social engagement, lack of initiation of interactions, deficits in communicative gestures and language, poor eye contact, severely impeded capacity to symbolize, and absent play. “Child A” (6 years old) displayed excessive acrobatic mobility, oral self-stimulation, high frequency of echolalia, and absent use of words for communication. “Child B” (5.5 years old) began the group unweaned, with no spoken language, characterized with highly inflexible thought and behavior and consistent facial self-stimulation, shifting between social disengagement and absolute dependency on others. “Child C” (4.5 years old) presented repetitive undifferentiated exploratory behavior, spent a lot of time dropping objects, falling or crashing into objects; he had not developed language skills, and the level of language comprehension was unclear.

### Group Setting and Procedure

The group met over a course of 30 weekly 45-min sessions. Group meetings were conducted in a room inside an ASD kindergarten and comprised 4 phases: #1 opening song, #2 drawing on paper (~20 min), #3 free movement with stretch bands (~20 min), and #4 closing song. A visual symbol of the group was created and placed in the personal schedule of each child, a strip of Velcro on which visual cards of the daily routine are placed. We also prepared a strip of cards representing the sequence of the group. Notably, visual pictures are commonly used with individuals with ASD, designed to increase predictability, prompt information processing, filter and clarify stimuli, embed cognitive sequences, and enhance understanding and cooperation (Quill and Institute, 1997; Vaz, 2013). Moreover, the high functional connectivity found between visual cortical areas of the brain and between visual and motor regions highlights the key role of visual transfer in visuo-motor activity (Barbeau et al., 2015).

Props brought in by the therapists were two colorful stretch bands (8′ diameter and 12′ diameter), three sheets of paper (33.11′ × 46.81′), and a small basket with colorful oil pastels. The choice of crayons was based on familiarity with the material, expressive potential, and ease of use with a broad range of both “clean” and “messy” work, also enabling a sense of control (Moon, 2011). A CD player was placed on a shelf; we used a constant music disk with various instrumental melodies for core group activity (phases #2 and #3). Other toys and objects in the room were taken out or covered, in order to minimize distractions.

Prior to the group meetings, we spread the two bands on the floor in an outer and an inner circle, sheets of paper and crayons were prepared at the side of the room. Upon entrance, after removing their shoes, the group sat inside the circle created by the large band, holding the smaller one. The group started and ended by counting “1–2–3,” followed by an opening/closing song, with a similar tune. The songs included simple wording, prompting movement of the upper torso, arms and head from side to side, up and down, stretching and squeezing, using rhythmic synchrony of song and action. The songs also included names, acknowledging the group as a whole and the participants in it, verbally and with pointing gesture. Afterward, we presented the visual symbols of the group sequence, turned on the music and spread the sheets of paper on the floor, making the crayons accessible for use. While moving to the next phase, the sheets of paper were attached to the wall using Velcro strips (prepared in advance on the back of the sheets), to become a part of the room as the group’s physical container. Next, we brought out the stretch bands again for free spontaneous use. A “Goodbye” closing song ended the group session. Following each session, our collaboration as co-therapists manifested in shared reflection and evaluative processes.

### Co-therapists: Shared Holding of the Group Container

As mentioned, the group was co-led by an occupational therapist and a dance/movement therapist (the author). Co-therapy, a common practice in group interventions, enriches both group members’ experience and the group process (Yalom and Leszcz, 2005). Advantages of a co-led ASD group included the modeling of communicational processes, learned from the interaction between therapists. We could offer alternative professional perspectives, enriching intervention strategies and later a reflection process, which help “read” the enigmatic behavior of these children and share with them the understanding of their emotional states (Tustin, 1992; Schore, 2012). We mirrored the children’s motor intentionality, and adequately handled the props to support their use, while being empathetically attuned to explicit and implicit expressions and communicative intentions, initiation, and responsiveness in the group. Importantly, the shared holding of the sequence of events, while weaving and at times creating intra- and interpersonal links, contradicts the autistic impairment of breaking the whole into parts and details (Happé and Frith, 2006) and impaired organization of motor chains underlying action representation (Cattaneo et al., 2007).

A meaningful aspect of co-therapy is the partnership not only in the liminal “potential space” but also when encountering challenges of autistic flattening to a “two-dimensional” quality of experience. Countertransferential awareness was critical in order to differentiate between progressive responsive acts in the group as opposed to repeated defensive ones. Upon experiencing the vanishing of an “inner” depth dimension, the appearance of autistic defenses as sensory-oriented maneuvers upheld feelings psychodynamically described as the evacuation of meaning and an emptying of relations (Meltzer, 1975; Alvarez, 1992; Tustin, 1992). Empathically attuned to the child’s signaling, we allowed moments of withdrawal and then, with creative “live company,” become more active in “reclaiming” him back into connectedness (Alvarez, 1992). Alvarez theorized this “vitalizing” form of intervention, in which the therapist actively reaches out to contact and “reclaim” her inaccessible patients, engaging them in the world of emotions and relationships (Alvarez, 2012). Aware of fine lines preserving the shared movement and utilizing the creative props, we referred the child’s attention to what was happening in the group, inviting him to join in, encouraging mutual interest, and promoting the choice to return and communicate.

The co-therapeutic relations evolved with progression of the group process (Dugo and Beck, 1997), from initial creation of our bond, definition and clarification of the group aims and setting of norms, to the construction of a cooperative team and formation of team identity, offering mutual support built on trust and complementarity. Our roles in the group became more fluid and balanced. Together we could identify shared states, leading the development of group rituals; we became familiar with the individual and group patterns and potential flexibility within the group as well as limitations.

## Clinical Vignettes

In this section, descriptions of the group process, including predominant themes and behaviors, will be presented in four vignettes, according to the developmental sequence of the group.

### The Beginning: Group Fragments

The group initiation stage was characterized by an experience of disorganization, unrelatedness, and high vigilance, while aiming to hold together the undifferentiated “pieces.”

The children were escorted to their daily schedules and then into the room. In the opening song we held the band by placing our hands on top of theirs, when we released the support they quickly loosened their grip. “Child A” would get up in the middle of the song to move around us in constant mobility. We acknowledged his presence and he transiently turned to look at us. In transition to the next phase we spread the sheets of paper and brought out the box of crayons. “Child A” lay down, at times grabbing a crayon and trying to peel off the paper wrapping or put it in his mouth. When we approached him he got up to move again in cartwheels in an overflowing movement. “Child B” was busy arranging the crayons in a line and then drawing distinct lines of color below. Gradually, we could join him, only to draw additional lines in his row. “Child C” was lifting and dropping crayons, vocalizing his thrill from the falling objects. When we put our hand on top of his, moving the crayon, he looked at the appearing color and would transiently continue the movement by himself. Our attention to others triggered “Child A’s” response, once deliberately bumping into another child, we stopped him for recognition of his need and clarification of the group rule to avoid harmful behaviors, “you are here with us, you want to be seen, we see you, but this is not allowed.” This child also presented occasional aggressive responses to physical touch, a response that decreased over time as physical proximity became more familiar and less aversive in the group.

When we replaced the sheets of paper with the stretch bands, they were first explored sensorially. We shifted between handling the bands, mirroring, accentuating rhythmicity of the music, while the children showed no clear intention to adjust motor actions to the others. Then a circular movement began, as children turned around themselves and the band started tightening around their body. The three moving bodies spun around. While handling the band, we recognized the appearance of joining and clashing patterns among children, children briefly looked at each other, began turning toward one another, then losing their balance to fall down and get up again for “another round,” smiling. Afterward, we acknowledged the joint positive affect while untangling their body from the wrapping bands.

### Phase II: Boundaries and Shared Space

With the progression of the group process, the children began to hold the band on their own in the opening and closing circle, while participating in movement, imitating the therapists. They also began pointing while naming group participants in gesture-word combinations. During the drawing phase “Child B” spread his hand on the paper, trying to draw its outline. Then, handing us the crayon, the marking of his hand drew the attention of the others and we began to mark other hands leaning on the paper. Next, while attaching the paper to the wall, we acknowledged the different hands that were presented.

When bringing in the elastic bands for free movement, the spinning motion that developed spontaneously, with increased intent to move toward others, enabled and advocated proximity. The children then began stretching the band in different directions, intentionally and unintentionally, transiently facing others to pull it against a therapist or another child. In times of a child’s disengagement, we could use the band, proffering the object to him, re-collecting him back into contact. At the end of the second month of group work, we experienced one of the miracle moments that was created and re-created, the establishment of a group circle, in which all participants took part using the stretch band. The circle held on while the children could step out or run around and return to hold the band within the group arena, allowing occasional eye contact, with a rising expression of amusement and feeling of togetherness.

### Phase III: Movement into Experiential Space

As group process advanced, the children more easily gathered near their schedules and we entered the room together. “Child A” could now stay with us throughout the whole opening song, partly lying down, “Child C” moved according to the rhythm and “Child B” already knew the lyrics. As we put down the sheets of paper, the children willingly spread their hands or feet for us to color around them, occasionally involved themselves in the marking of their own or another’s hand or foot. When “Child A” lay down on top of all three sheets, we drew around his whole body. Following this, he got up to look at his body outline and the other children were then motivated to imitate him and lie down for the same purpose. “Child B” also began drawing round forms and we could join him for a scribbled dialogue, with joint attention of the others and transient active participation in drawing.

Of note, as mentioned earlier, there was occasional disconnection and disengagement of a child from others, for instance by adhering to the surface of the floor or walls of the room, requiring an attuned offer of a vitalizing, reclaiming function.

During the free movement, the children tried out different ways of moving with the band—stretching, pulling, hopping over it—while imitating each other. They also responded to changes in the rhythmic pattern of the music. We emphasized this by vocalizing the rhythm, bouncing and swaying, clapping and tapping on the body. At one point “Child B” hopped over the band, looked at us and said “turn,” we started waving the band around him as a jump rope, vocalizing enthusiastically the preparation and actual jump, while the other children joined in; “my turn,” said “Child A.” When we later stretched the band between the two of us, the children began running inside this zone, in turns, to be held by one therapist and then the other. “Child C” also initiated passage while stepping on the band, from side to side, an action that also motivated the joining of the others. We were now witnessing a group instigated to move together.

### Phase IV: “Relay Station” for Connectedness

Anticipation for group meetings arose, the children checked to look for the group visual symbol in their schedules, also approaching us in the morning to check that we were meeting later.

While drawing, the children were engaged in the outlining of their body parts, partly filling the shapes with color. “Child A” once stood on his head on the paper. One therapist then held his feet while the other drew a circle around his head. The two other children, to our amusement, moved to do the same. The children initiated this in the next sessions as well.

Experimental movement with the bands enhanced, practicing variations of activities that became familiar, e.g. using them as a jump rope or for passage in-between, stretching to create a group circle. “Child B” once began running around us in circles, we spontaneously presented our palms for him to touch as “relay stations.” This triggered the others to join him, while order was maintained so the children alternately touched our hands, without bumping into each other, in a rhythmic flow, with facial and vocal expressions of thrill. A dynamic dance momentum developed within the playful atmosphere.

In the final stage of the group process, children cooperated more in the structured opening and closing phase, eye contact was more prominent and in the open parts of the group sequence children were more initiative and responsive to others. Events such as those aforementioned that were initiated and driven by the children became our shared rituals.

## Discussion

This manuscript presents group work with young children with autism who are minimally verbal and have diverse developmental and communicative deficits. In a dynamically oriented creative arts intervention we intended to promote spontaneous shared experiences, expanding expression and communication mostly *via* nonverbal means. A playful environment was created through the accumulation of embodied relational experiences, setting in motion actions and reactions in the group, whereby children initiated interaction, leading and following group movement, according to the occurrences in the “here-and-now.” The group process is hereby discussed with reference to three intertwining axes of the therapeutic work: the first axis characterizes the use and implementation of the nonverbal creative means; the second delves into the physical and mental developments established through sensory and motoric experiences; the third focuses on the development of connection and communication in the group.

### Use and Implementation of Creative Nonverbal Means

#### Drawing With Oil Pastels

The functional and social use of crayons developed throughout the sessions. At first, the focus of the children was mainly on the sensory qualities, treating the crayons as autostimulating or autistic objects (Tustin, 1992). This autosensual use did not vanish but diminished over time. The drawing of the body outline was initiated by choice and created with cooperation of the children; what began as an act of one expanded, promoted mimicking, and became a shared group experience. Marking of the body shape, as a personal signature of each child, holds a potential contribution to the body image and sense of self (Steinhardt, 1985). Notably, in most cases the children lay down on their stomach, presenting their back, the rear facade, which is more sealed and bony, avoiding eye contact and protecting their front facade which is soft and full of “windows” (Pollak, 2009); toward the end of the year children began turning over to expose their frontal communicative body.

The body outline drawing, and also occasional scribbling, creating lines and abstract forms in turn, enabled joint attention and action, i.e. coordination of attention with others. This indexes the understanding of the other as an intentional agent. Simultaneously, the three separate paper sheets were joined to form a shared drawing space. We note the meaningful placement of the product on the wall for the children to see, becoming part of the containing group space.

#### Movement With Shared Elastic Bands

In the transition to the next phase, the stretch bands were brought back into the “center of the arena.” Use of the bands first and foremost supported the holding of the “group fragments,” making contact, creating linkage and also resonance to their movement. Inside the tangle of the band that the children were motivated to create, they might have experienced and displayed the fantasy of being in the same envelope together with someone else, looking out at the world. Gradually, exploratory movement increased, by stretching, bending, twisting and turning, binding the band around the body and pulling it strongly. This expansion of movement repertoire was carried out in the vertical plane (upward and downward), against gravity; in the horizontal plane—exploring the shared space around; and in the sagittal plane—toward and away from others, representative of interpersonal responses (Loman and Merman, 1996).

The band, as an elastic border, had crucial contribution to a meaningful moment in the group—the creation of a circle. The group circle was a construct created during spontaneous movement by all participants, eliciting temporary eye contact as well as mimicking and experiential placement changes. The band later supported the enactment of creative ideas initiated by the children, in a playful field of uncertainty and surprise. Therefore, the prop (as well as accompanying music and rhythm discussed below) may have operated as a transitional object (Winnicott, 1953), something “other” than the child providing a projective distance necessary for him to feel safe to socially interact. The structure or form itself became a vehicle for discovering the creative surprises that liberated the mind at play (Nachmanovitch, 1990).

#### Instrumental Music and Rhythmicity

Background music was used to create a sensory and emotional “envelope” that is continuous, diverse, and non-intrusive. The lack of lyrics in the melodies chosen also aimed at avoiding masking of spontaneous vocality in the group. At first, we were preoccupied with adjustment of volume to a tolerated level and avoiding distraction. Gradually an interest in the music source appeared, then responses to change in tempo, followed by the creation of rhythmic group movement. Importantly, music has been shown to promote mutual emotional understanding in children with autism (Katagiri, 2009), with relatively intact perception and processing of music-evoked emotions in ASD (Caria et al., 2011).

Rhythmicity was created in the group, alongside the rhythm of the music, by clapping, patting, swaying, and vocalizing movements. Indeed, rhythm is one of the valued elements in creative psychotherapy groups, enabling transition from chaotic experiences to an initial external order that may prompt inner organization, forwarding integration as well as relational processes and communication (Schmais, 1985; Chaiklin and Schmais, 1993; Trevarthen and Fresquez, 2015). Rhythm serves developmental functions of separation and boundary formation (Loman, 1998). The significance of joint rhythmic movement in the group is drawn from the suggested role of rhythm and timing in ASD and its use as scaffolding to build social and communicative interactions (Amos, 2013; Hardy and LaGasse, 2013). This may also be in line with Tustin’s “rhythm of safety,” a mind–body state in which sensory experiences and individual bodily rhythms become re-associated with a relational, cooperatively founded tempo involving self and other (Mitrani, 2010).

### Psychophysiological Processes Set in Motion *via* Sensory-Motor Activities

Contemplating the sensation-dominated nature of the autistic experience (Tustin, 1992), evidence regarding the importance of sensory perception and modulation in ASD continues to accumulate, as a major contributor to the symptomology, with particular relevance to social contexts (Baranek et al., 2006; Ben-Sasson et al., 2009), now also a diagnostic criterion (American Psychiatric Association, 2013). The versatility of sensorimotor experiences in the presented group, supported by rhythmicity, triggered complementary signals from the individual’s body and surroundings, influencing central integration. A discussion of the dynamic and reciprocal interactions between the physical and the mental developmental aspects that were promoted through the group process follows.

#### Tactile Contact

Throughout the group sessions, the children experienced different kinds of touch, active and passive, as an outcome of intended and random meetings, at times *via* the prop (the band) that mediated indirect rather than direct human contact. The tactile system, a barrier between the inner environment and the surroundings, depends upon the co-activation and balance between its two components, the discriminative system and the protective system, to decipher and respond to tactile information (Parham and Mailloux, 2005). Abnormal tactile sensitivity is prevalent in individuals with ASD, with highly heterogeneous responsiveness (Marco et al., 2011). In line with Anzieu’s concept-theory of “Ego-skin” and constitution of “psychic envelops” (Anzieu, 1989) and Ogden’s “autistic contiguous position” and “sensory floor” (Ogden, 1989), intersubjective experiences that combine sensory skin input, while providing a containing function, are the fundamental means for creating mental meaning and the construction of the self. These have become distorted in the polarized autistic state, characterized by confusion between inner and outer environment, lack of a containing and unified body envelope, and a fixated world of sensuous self-stimulation (Tustin, 1992). Children with ASD were recently shown to respond to affective touch with atypical hypoactivation of the “social brain,” i.e. a network of brain regions shown to be involved in social–emotional information processing, also marking tactile response as a target for measuring improvement in intervention studies (Kaiser et al., 2016). Notably, a previous study indeed demonstrated the reduced touch aversion following group dance/movement therapy with young children with autism (Hartshorn et al., 2001), thus, pointing to the importance of adequate tactile relational exploration.

Indeed, we witnessed situations in the group whereby touch was experienced as aversive, threatening, or confusing. For “Child A,” proximity triggered aggressive behavior at first. Opportunities for safe touch accumulated during the group process, while we were actively attuned to the children’s response to the closeness of another, setting group roles and boundaries. As the group progressed, children began allowing and seeking proximity. Adequate tactile input in a shared therapeutic setting promotes clarification of body boundaries and enables exploration of the relation between the body and the environment, thus promoting early body organization, development of body image and initial self-other relations (Erfer, 1995; Samaritter and Payne, 2013).

#### Inner Vestibular and Proprioceptive Sensation

Motor movement that developed during group sessions enabled changes in placement and movement against gravity, by which the vestibular system came into play. A sense of effort and force was also provided, which augmented proprioceptive input. The vestibular system yields sensory input for balance, spatial orientation, and gravity, while the proprioceptive system yields awareness of body position and an interior sense of the relational positioning of body parts; both have been shown to contribute to atypical sensory experiences in ASD (Bogdashina, 2016). Moreover, the autistic brain was shown to build an abnormal association between self-generated motor commands and proprioceptive feedback (Haswell et al., 2009).

Stern links proprioceptive feedback to the construction of the core-self, postulating that the instilling of a sense of agency and subjectiveness is founded on a sequence of processes; these include the sense of volition prior to the motor action, the proprioceptive feedback during movement, and the predictability of its outcome (Stern, 1985). During group sessions, we witnessed the lack of such sequence when an action was performed randomly or without meaning. Yet, with time, throughout the group process, we noticed and could acknowledge the appearance of intent and will, the motivation behind the action, and the anticipation of its result. Thus, group body-related experiences provided a sense of the movement’s meaning, forming elementary actions of relating (Samaritter and Payne, 2013).

#### Sensory-Motor Integration

The group became an arena for the interplay between the personal unique sensory world of each child and the interpersonal experience, as opposed to the autistic self-stimulation that serves to isolate oneself from external social engagement. Tactile sensations and complementary gravity-based data coming from inside the body *via* the proprioceptive and vestibular systems support the formation of a mental structure of the self in object-related contexts (Pollak, 2009). This relies on sensorimotor integration, shown to be disrupted in individuals with ASD (Marco et al., 2011). The emergence of inner representations of the body and the outside environment arises from cross-modal sensorimotor integration, critically involved in the construction of the body scheme (Maravita and Iriki, 2004; Shenton et al., 2004). In the current group, the use of multiple channels of communication *via* sensorimotor experiences upheld the perception, interpretation of information, and enactment of adequate responses. Cross-modal attunement to the children’s “vitality” affect, i.e. the empathic attuned response in movement and vocal sounds, had a central role in emphasizing the emotional quality of the experience and a sense of “aliveness,” promoting emotional co-regulation (Stern, 1985).

Group work thus facilitated both “bottom-up” processes, i.e. from the sensory-motor level to the higher mental processing and the creation of meaning and will, and “top-down” processes, i.e. from the children’s intentions, anticipations, and memories to the sensory-motor bodily response.

### Seeds of Connection and Development of Communication in the Group

The establishment of the group as a holding and facilitating environment, after the settling-in period, was recognized first and foremost by the emerging motivation to participate.

Moments of “state sharing” emerged in the group (Stern, 1983), dialogues of shared sounds and body movements. These provided a sense of form and boundedness, so that the children could feel their own edges and not meld into the other or retreat into the confines of their solitary enclave. Authentic moments of shared experience may be characterized as “moments of meeting” (Stern et al., 1998), during which, in a space of uncertainty, a mutual understating emerged among group participants while “moving along” in the “here-and-now.” Given the adequate attunement and response of the therapists, this nonverbal communication revealed a novel way of being together in the interpersonal environment, establishing mutual regulation and a basis for implicit knowledge of relations.

With time, children were more explorative, took more initiative, and became familiar and cooperative during transitions in the sequence of the meeting. Moreover, we could see enhancement in awareness and acknowledgment of others in the group. A profound development throughout the group process was the enhancement of mimicry. Ogden highlighted the manner in which Winnicott used to value and conceive the role of imitation as a primitive form of object relatedness, also emphasizing imitation as a phenomenon pertaining to psychic functioning in the generation of subjective experiences (Ogden, 1989). Substantial research shows impaired imitation in individuals with ASD (Rogers et al., 2003; Edwards, 2014) and deficits in interpersonal synchronization (Marsh et al., 2013; Fitzpatrick et al., 2016), pointing to the importance of mimicking behavior of the children in the group, as grounds for social communication and mutual play (Field et al., 2001).

Along with the growing ability to spontaneously imitate others’ actions, movement became more deliberate and intentional, shifting the predominant group patterns. Dynamics of change in distance from one another were apparent, corresponding with the motivation to seize opportunities to connect while taking the risk of making contact (Shahar-Levy, 2009). The significance of these possibilities for alterations in interpersonal distance and proximity to others derives from the abnormalities shown in physical relational distance and personal space in individuals with ASD (Asada et al., 2016).

With further advancement and development of the group, “moments of meeting” accumulated and the children were capable of sustaining longer interactions. Children’s behaviors aimed at influencing each other appeared, with persistence until achieving the intended response or registering failure. Prolonged interactions enhanced waves of awareness on which the group narrative was constructed. Familiar group rituals were created, involving playfulness, joint action and mutual joy, promoting initial representations of relationships. Examples of group activities included drawing of the body outline and dynamic group movement patterns: the creation of a group circle with the stretching of the bands; using the band as jump ropes; spinning around in front of each other while the bands tighten around the body; passage between therapists inside the zone created by the band; running around the therapists acting as “relay stations” for group movement. Such patterns of group movement initiated by the children demanded acknowledgement of the others and spatiotemporal organization. Through the facilitation of group work, objects that were first static obtained unlimited qualities that led to the development of new associative networks, as happens in imaginative play.

Group reoccurring activities were founded on the interconnection between the explicitly known remembered elements (autobiographic memory), alongside the nonverbal procedural memory (Siegel, 2001). The remembered shared moments in the group were particularly important due to ASD research showing specific deficits in relational memory (Maister et al., 2013) and impaired autobiographic memory for personally experienced events (Bruck et al., 2007; Crane and Goddard, 2008; Bordigon et al., 2015), shown to be reciprocally related with the sense of self (Lind, 2010). Connections emerged, otherwise difficult to ignite.

To conclude, the paper presents a model of group therapy for children with ASD, in a dynamic constellation that mainly focused on nonverbal creative means of expression and communication. The group provided a defined yet flexible space for spontaneous explorations and responsiveness, where uncertainty challenged the need for predictability, enabling the emergence of social engagement through creative play. The group process highlighted the reciprocal developmental relations between physical and mental movement. In the participating children, who initially did not play or talk, motivation to connect and be seen was revealed and realized. By doing so, personal and group processes were set in motion. Seeds of curiosity were discovered and planted, fertilized and began to grow, encouraging further application of creative psychotherapeutic strategies for working with children with autism in group settings.

## Author Contributions

SV prepared the manuscript, reviewed the literature and wrote the paper.

### Conflict of Interest Statement

The author declares that the research was conducted in the absence of any commercial or financial relationships that could be construed as a potential conflict of interest.
